# Improved HbA1c and reduced glycaemic variability after 1-year intermittent use of flash glucose monitoring

**DOI:** 10.1038/s41598-021-03480-9

**Published:** 2021-12-14

**Authors:** Wenhui Zhang, Yu Liu, Baosheng Sun, Yanjun Shen, Ming Li, Lanbo Peng, Honggang Duan, Xudong Su, Shaoxia Lu, Xiaoqin Tian, Yaqiang Tian

**Affiliations:** 1 Department of Endocrinology, Liaocheng People’s Hospital, Liaocheng, 252000 China; 2 Cadre Health Care Department, Liaocheng People’s Hospital, Liaocheng, 252000 China; 3 Department of Endocrinology, Liaocheng Veterans Hospital, Liaocheng, 252000 China; 4 Department of Pulmonary and Critical Care Medicine, Liaocheng People’s Hospital, Liaocheng, 252000 China; 5 Department of Endocrinology, Liaocheng Central Hospital, Liaocheng, 252000 China

**Keywords:** Diseases, Endocrinology, Medical research

## Abstract

Flash glucose monitoring (FGM) was introduced in China in 2016, and it might improve HbA1c measurements and reduce glycaemic variability during T1DM therapy. A total of 146 patients were recruited from October 2018 to September 2019 in Liaocheng. The patients were randomly divided into the FGM group or self-monitoring blood glucose (SMBG) group. Both groups wore the FGM device for multiple 2-week periods, beginning with the 1st, 24th, and 48th weeks for gathering data, while blood samples were also collected for HbA1c measurement. Dietary guidance and insulin dose adjustments were provided to the FGM group patients according to their Ambulatory Glucose Profile (AGP) and to the SMBG group patients according to their SMBG measurements taken 3–4 times daily. All of the participants underwent SMBG measurements on the days when not wearing the FGM device. At the final visit, HbA1c, time in range (TIR), duration of hypoglycaemia and the number of diabetic ketoacidosis (DKA) events were taken as the main endpoints. There were no significant difference in the baseline characteristics of the two groups. At 24 weeks, the HbA1c level of the FGM group was 8.16 ± 1.03%, which was much lower than that of the SMBG group (8.68 ± 1.01%) (*p* = 0.003). The interquartile range (IQR), mean blood glucose (MBG), and the duration of hypoglycaemia in the FGM group also showed significant declines, compared with the SMBG group (*p* < 0.05), while the TIR increased in the FGM group [(49.39 ± 17.54)% vs (42.44 ± 15.49)%] (*p* = 0.012). At 48 weeks, the differences were more pronounced (*p* < 0.01). There were no observed changes in the number of episodes of DKA by the end of the study [(0.25 ± 0.50) vs (0.28 ± 0.51), *p* = 0.75]. Intermittent use of FGM by T1DM patients can improve their HbA1c and glycaemic control without increasing the hypoglycaemic exposure in insulin-treated individuals with type 1 diabetes in an developing country.

## Introduction

Type 1 diabetes mellitus (T1DM) is a challenging chronic autoimmune condition resulting in a complete cessation of insulin production. Every year there are an estimated 13,000 new T1DM patients diagnosed in China. Most of them are adults treated with multiple daily injections (MDI) of insulin^1,2^. There are some unique clinical and demographic characteristics among Chinese type 1 diabetes patients, such as poor blood glucose control, extremely low blood glucose monitoring frequency, irregular insulin treatment, frequent acute and chronic complications, a late onset and a lean body habitus^3^. It is necessary to achieve favourable glucose levels for T1DM patients to prevent diabetes-related complications, including severe hypoglycaemia, life-threatening diabetic ketoacidosis (DKA), macrovascular complications (such as peripheral arterial disease, coronary heart disease and cerebrovascular disease) and microvascular complications (such as retinopathy, nephropathy and neuropathy)^4^.

As the basis of modern T1DM treatment, blood glucose monitoring has played an important role for many years. In 1978, self-monitoring blood glucose (SMBG) was first applied in clinical therapy^5^. The close relationship between SMBG frequency and improved diabetes control has been confirmed by several studies^6^. However, there are many problems with SMBG, including stress, cost, pain and the required technical skills, which have led to the development of retrospective continuous glucose monitoring (CGM) and real-time continuous glucose monitoring (rt-CGM)^7^. CGM can provide information that SMBG cannot obtain, such as a real-time display of glucose levels and glucose change rates and the ability to characterize blood glucose variability^8^. Randomized controlled trials have found that CGM combined with subcutaneous insulin infusion (CSII) or multiple daily injections (MDIs) could decrease haemoglobin A1c (HbA1C) levels and reduce hypoglycaemia^9–12^. In recent decades, remarkable progress has been made in the management of T1DM, partly due to CGM and rt-CGM technology^6,13^. Although the accuracy and usability of rt-CGM have been gradually improved, this technology has not been widely adopted thus far because of its cost and inconvenience^7,8^.

In 2014, a new continuous glucose monitoring system-flash glucose monitoring (FGM) was introduced to the European Union, and then it was available in China in 2016^14^. There are some major differences between FGM and other real-time CGM systems. FGM can provide an ambulatory glucose profile (AGP) for 14 days without any need for blood calibration, which has attracted the attention of clinicians and people with diabetes.

In this study, we divide the patients into the FGM group or the SMBG group and carry out a 50-week single-site randomized controlled trial to compare the differences of HbA1c, TIR, duration of hypoglycaemia and the number of diabetic ketoacidosis events between the two groups. Our overall goal is to investigate whether the use of FGM could be an effective method for achieving improvement of HbA1c and reducing glycaemic variability during T1DM maintenance.

## Methods

### Study designs and aims

**Figure 1:**
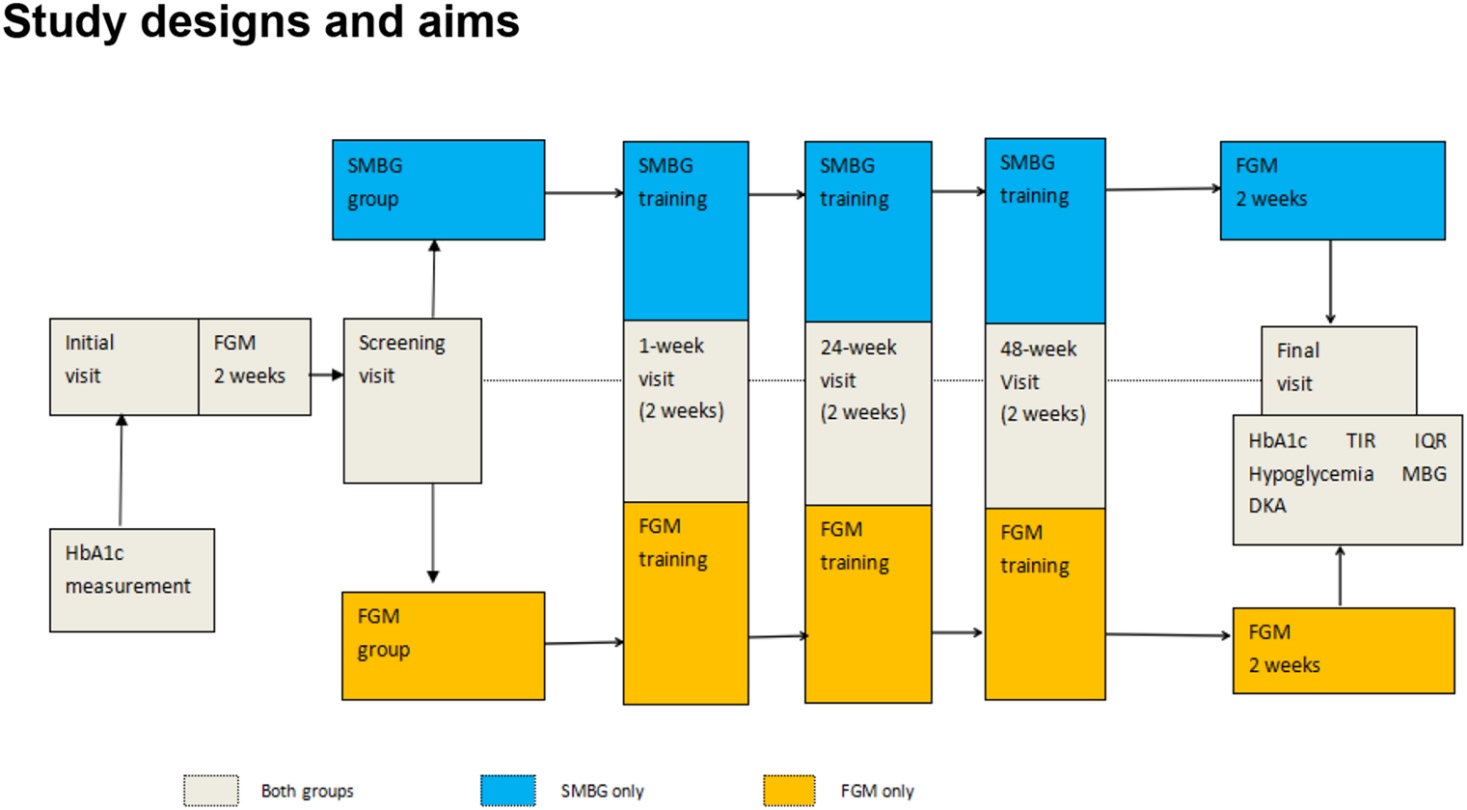
Trial design.

We carried out a 50-week single-site randomized controlled trial to evaluate the effect of FGM on blood glucose control and variability in T1DM patients with MDI therapy (Fig. 1).

### Sample size

The estimation of the sample size was based on the estimated incidence of T1DM in China and the results of STAR3 trial^2,15^. A decrease in HbA1c by 0.3% is generally considered a clinically significant decrease to reduce diabetic complications. With a power of 90% at *P* = 0.05 (two-sided), 72 patients in each group are necessary to detect a 0.3% difference in HbA1C between the two groups. Therefore, this study needed 160 patients, who were selected from Liaocheng People's Hospital, Liaocheng Veterans Hospital and Liaocheng Central Hospital, assuming a dropout rate of 10%.

### Population

We are trying to establish a T1DM management model in underdeveloped areas. Our hospital serves a catchment area of approximately 10 million people, and our group has been devoted to the registration and management of baseline data for T1DM patients in the Liaocheng area over the past six years. We have determined the prevalence of T1DM in Liaocheng in a previous study, which is consistent with that in Guangdong Province^3,16^. In this study, all of the participants were recruited from our management system, and had received regular and systemic diabetes education.

The inclusion criteria were as follows: (1) age ≥ 4 years old; (2) HbA1c ≥ 7%; (3) requiring multiple daily injections (MDIs); and (4) a diagnosis of T1DM for at least 3 months.

The exclusion criteria were as follows: (1) patients with severe complications; (2) pregnancy; and (3) application of FGM and/or CSII in the previous 3 months.

This study has been registered in Chinese clinical trials as ChiCTR-INR-16009665(10/27/2016). In addition, the clinical research program was approved by the Ethics Committee of Liaocheng People’s Hospital. Written and verbal informed consent were obtained from all participants, including those of children under 16, which were obtained from their parent. All experiments were performed in accordance with relevant guidelines and regulations.

### Study procedures

#### Data collection

We collected information on patient age, diabetes course, insulin dosage, and calculated the BMI of the participants. After the HbA1c analysis was performed, all of them wore a FGM for 2 weeks. Data including glucose fluctuations, time in range, mean glucose, and hypoglycaemia time were downloaded and calculated as the baseline during the 1st week without any intervention. Then, the patients were randomly divided into the FGM group or the SMBG group at a ratio of 1:1 by table of random number.

#### Procedures

Both groups wore the FGM for multiple 2-week periods, beginning with the 1st, 24th, and 48th weeks for gathering data, while blood samples were collected for HbA1c measurement. We gathered baseline data in the 1st week. When the participant scanned the sensor with the reader, he could get current sensor glucose level, a glucose trend arrow, and glucose readings for the previous 8 h. While putting the reader close to the sensor for a few seconds, the participant could get the 14-day glucose profile. In the 2nd week, the FGM group received directions on the best use of FGM, including meanings of the trend arrows and glucose profiles for treatment adjustment, and how to deal with hyperglycaemia and hypoglycaemia. Patients of FGM group were required to perform at least 7 scans per day (before and after meals and before bedtime). At the same time, the SMBG group underwent 3–4 blood glucose measurements daily, including an assessment of FPG and post-meal measurements. The SMBG group could not view results of the 14-day memory glucose profile since they were not provided a handheld reader. Dietary guidance and insulin dose adjustments were made for the FGM group patients according to their Ambulatory Glucose Profile, and for the SMBG group patients according to their SMBG measurements taken 3–4 times daily. From 3 to 23 weeks, the SMBG measurements (3–4 times a day) continued, and routine care from our management system was received by both groups. Both groups wore the FGM device again at 24 to 25 weeks, receiving the same interventions as before while gathering data, and blood samples were collected for HbA1c measurement. Then, from 26 to 47 weeks, all of the participants underwent SMBG measurements and interventions on the days when not wearing the FGM device.

#### Outcomes

The follow-up phase began at 48 weeks. All of the participants were required to wear the FGM for 14 days again. The data were downloaded and blood samples were collected for HbA1c measurement at the final visit. HbA1c, TIR, duration of hypoglycaemia and the number of diabetic ketoacidosis events were taken as the main endpoints.

### Statistical analysis

SPSS statistical software version 17.0 was used to perform the statistical analyses. Normally distributed continuous data are presented as the mean ± SD, and skewed continuous data are summarized as medians. For categorical data, we used numbers and proportions for analysis. All of the data were tested at the 5% significance level. A t-test was applied to analyze normally distributed data and the Wilcoxon signed rank test was used to analyse skewed continuous data. In addition, the chi-squared test was used to assess differences in proportions.

## Results

### Clinical characteristics

A total of 160 patients were recruited from October 2018 to September 2019 in Liaocheng, Shandong Province. Because of refusal to participate in the study, 146 subjects (64 men, 82 women) were only analysed at baseline. As presented in Table 1, there was no significant difference in the baseline characteristics of either group, including age, course of disease, body mass index and insulin dosages after randomization (*p* > 0.05). The baseline blood glucose characteristics (HbA1c, TIR, IQR, MBG and duration of hypoglycemia) in the first week without intervention described in Table 2 also showed no significant differences.

**Table 1:** Baseline characteristics of participants [${\overline{\text{x}}}$ ± s, M(QL, QU) , n/n].

Group	n (M/F)	Age (Years)	DM duration (Years)	BMI (kg/m^2^)	Insulin dosage (u/(kg.d))
FGM	71(34/37)	36.68 ± 19.71	4. 0 (2. 0, 7. 0)	19.70 ± 2.01	0.68 ± 0.08
SMBG	75(30/45)	35.19 ± 18.91	5. 0 (2. 0, 7. 0)	19.13 ± 1.66	0.69 ± 0.07
p-value	0.34	0.64	0.67	0.06	0.43

FGM: Flash glucose monitoring; SMBG: Self-monitoring blood glucose; BMI: body mass index.

**Table 2:** Baseline blood glucose characteristics of participants (${\overline{\text{x}}}$ ± s).

Group	HbA1C(%)	TIR(%)	IQR(mmol/L)	MBG(mmol/L)	Hypoglycemic duration(min/d)
FGM	9.05 ± 1.43	36.49 ± 17.57	8.39 ± 2.69	11.79 ± 2.20	201.96 ± 44.28
SMBG	9.07 ± 1.18	37.87 ± 15.87	8.10 ± 1.94	11.81 ± 1.93	198.13 ± 35.90
p-value	0.95	0.62	0.46	0.95	0.57

TIR: time in range; IQR: interquartile range; MBG: mean blood glucose; Hypoglycemic duration: the time below 3.9 mmol/l.

### Comparison of glycaemic parameters at the 24-week and 48-week follow-up

As showed in Table 3, at 24 weeks, the HbA1c level of the FGM group was 8.16 ± 1.03%, which was much lower than that of the SMBG group (8.68 ± 1.01%) (*p* = 0.003). The IQR, MBG, and duration of hypoglycaemia of the FGM group also showed a significant decline, compared with the SMBG group (*p* < 0.05), while TIR increased in the FGM group [(49.39 ± 17.54)% vs (42.44 ± 15.49)%] (*p* = 0.012). At 48 weeks, the differences were more pronounced (*p* < 0.01).

**Table 3:** Comparison of glycemic parameters across 48-week study (${\overline{\text{x}}}$ ± s).

Group	n	HbA1C(%)	TIR(%)	IQR (mmol/L)	MBG(mmol/L)	Hypoglycemic duration(min/d)
FGM	71					
1 W		9.05 ± 1.43	36.49 ± 17.57	8.39 ± 2.69	11.79 ± 2.20	201.96 ± 44.28
24 W		8.16 ± 1.03	49.39 ± 17.54	6.85 ± 2.35	10.48 ± 1.63	175.86 ± 43.19
48 W		7.39 ± 0.71	62.35 ± 12.29	5.70 ± 2.03	9.21 ± 1.18	158.78 ± 35.31
SMBG	75					
1 W		9.07 ± 1.18	37.87 ± 15.87	8.10 ± 1.94	11.81 ± 1.93	198.13 ± 35.90
24w		8.68 ± 1.01*	42.44 ± 15.49*	7.57 ± 1.81*	11.22 ± 1.60*	191.95 ± 37.11*
48w		8.36 ± 1.02**	46.52 ± 16.65**	7.10 ± 1.91**	10.78 ± 1.62**	192.80 ± 31.08**

TIR: time in range; IQR: interquartile range; MBG: mean blood glucose; Hypoglycemic duration: the time below 3.9 mmol/l.

*: Compared with FGM at 24 W, p < 0.05; **: Compared with FGM at 48 W, p < 0.01.

For the FGM group, the HbA1c concentration decreased from 9.05% at baseline to 8.16% at 24 weeks (*p* < 0.05) and to 7.39% after 48 weeks (*p* < 0.05), resulting in an overall difference in HbA1c over the study period. At the same time, the IQR, MBG, and duration of hypoglycaemia also displayed marked decreases at 24 weeks and 48 weeks (*p* < 0.05). The TIR increased from 36.49 to 49.39% at 24 weeks to 62.35% by the end of the study (*p* < 0.05).

In addition, there was no observed change in the number of DKAs at the end of the study [(0. 25 ± 0. 50) vs (0. 28 ± 0. 51), *p* = 0.75].

With regard to safety, no sensor-related adverse events occurred and the few adverse events such as insertion and shedding problems were similar to the events observed in other studies^17,18^.

## Discussion

T1DM is an endocrine metabolic disease in which pancreatic islet β cells are destroyed by the autoimmune system, and insulin therapy is required. The management of chronic hyperglycaemia, hypoglycemia and blood glucose variability in T1DM is currently difficult in clinical treatment^19^. Monitoring glucose levels is essential for achieving target glycaemic control and avoiding hypoglycaemia, especially in patients with T1DM.

As a new technology, flash glucose monitoring (FGM) has recently been rapidly accepted by clinicians to replace CGM/SMBG. In FGM, glucose data could be stored for up to 8 h on a sensor and a handheld reader could be used to obtain them conveniently. Moreover, the FGM sensor is factory-calibrated and can be worn for up to 14 days, which improves the patients’ qualitiy of life^20^.

In this study, we aimed to identify whether using FGM can improve glycaemic control among patients with T1DM in Liaocheng District.

The 2017 "International Consensus of Continuous Glucose Monitoring" emphasized that the "three core indicators" of FGM monitoring are TIR, blood glucose variability, and hypoglycaemia. In 2019, Marion Fokkert collected daily life data from persons with DM using the FGM system and found that after a 1-year follow-up, their HbA1c declined from 64 mmol/mol to 60 mmol/mol. In addition, the patients reported increasingly less severe hypoglycaemia and a more active role in treatment^21^. The study of Ramzi A Ajjan et al.also found similar results. They reported that FGM can improve HbA1c and treatment satisfaction without increasing hypoglycaemic exposure in insulin-treated type 2 diabetes individuals managed in primary/secondary care centres^22^. Different from our research, they evaluated the Professional FGM blinded to the patients. What’s more, the results of a large randomized clinical trial known as IMPACT demonstrated the clinically significant reduction in HbA1c without increasing hypoglycaemic exposure in patients who were randomized to flash CGM, which is consistent with our findings^23^. The DIAMOND and GOLD studies also showed the benefit of CGM in people using conventional MDI treatment, with the majority of patients having T1DM^12,24^.

In our study, HbA1c was 9.05% at baseline, demonstrating the T1DM patients’ general poor glycaemic control in Liaocheng, while it decreased after intermittent use of FGM by 48 weeks (from 9.05 to 7.39%, *p* < 0.05), similar to the above studies. At the same time, their hypoglycaemic duration did not increase. Instead, it decreased sharply by the end of study, which demonstrated that FGM was able to improve HbA1c while also decreasing hypoglycaemic exposure.

Blood glucose variability, also known as blood glucose fluctuations, is an unstable state in which blood glucose levels change between peaks and troughs. The IQR is considered an appropriate value for expressing blood glucose variability^17^. IMPACT study demonstrated that there were apparent improvements in time in range and glucose variability in patients who were randomized to flash CGM^23^. In our research, we also observed obvious improvements in TIR at 48 weeks and a reduced IQR, which meant that patients in the intervention group achieved good glycaemic control and reduced blood glucose variability.

A study by Dunn et al. discovered that in real-world conditions, flash glucose monitoring with higher rates of scanning was linked to improved glycaemic markers, including increased time in range and reduced estimated HbA1c from 8.0 to 6.7%^25^. Research by Mingqun Deng also showed that the FGM-derived TIR could be helpful in the glucose management of Chinese adult T1DM patients, while glucose variability should also be taken into consideration in interpreting the relationship between TIR and HbA1c^26^. Our study obtained similar results: after intermittent FGM intervention, the TIR value increased from 36.49 to 62.35% at 48 weeks, and the HbA1c declined to 7.39%.

Many studies have shown that estimating glycaemic control from HbA1c alone can be misleading. Study of Beck et al. highlighted the discrepancy between average glucose and HbA1c on individual level^27^. A flash glucose monitoring system can provide real-time interstitial glucose levels and trends of glucose levels. Moreover, it has the advantage of being precalibrated, so the user does not have to perform any CBG^28^. Beyond that, the users could acquire a patient’s CGM glucose profile, which has considerable value for optimizing diabetes management.

Previous studies have all focused on the influence of the continuous application of FGM for T1DM patients. In China, this is the first study to discuss the effects of 1-year intermittent use of FGM in T1DM patients, which may be a useful strategy for follow-up studies and the application of FGM for diabetes prevention and blood glucose management.

This study has a number of limitations that should be noted. First, the participants could not be blinded to the intervention, which may affect the results. Second, the intermittent intervention could underestimate the potential benefit of FGM. Third, as some of the data including DM duration and insulin dosage were patient-reported, recall bias may be present. Finally, the current population was selected from patients in Liaocheng District, which may introduce selection bias.

## Conclusion

In summary, intermittent use of FGM in T1DM patients can improve HbA1c and glycaemic control without increasing hypoglycaemic exposure in insulin-treated type 1 diabetes individuals in an developing country.
